# Urotropin in the Lumbago of Febrile Affections

**Published:** 1908-10

**Authors:** 


### Urotropin in the Lumbago of Febrile Affections.

Franz Weitlaner (*Weiner klin.-Therap. Wochenschr., No.* 26, 1908,) adverts to the fact that some of the commercial hexamethylentetramins develop upon standing—especially when the air is moist—an odor of ammonia or formaldehyde which is offensive to the patients. This, however, he never saw with Schering's urotropin.

In 1904 (*Monatsh. f. prakt. Dermat.,* Vol. 39), he recommended urotropin in spinal neurasthenia with phosphaturia and lumbar fatigue and in all forms of lumbago. He now lauds it especially in influenzal lumbago and the lumbago which closely resembles it, *i. e.*, that occurring at the beginning of acute inflammations of the respiratory tract and angina. Here one or two urotropin tablets coup the affection or at least afford striking relief. Patients with lumbago that prevents their arising in the morning, whose every movement is painful, are able to go about in the room a few hours after taking urotropin.

Almost all febrile processes commence with stiffness of the extremities and lumbar pain. Here, too, urotropin acts beneficially. We must deposit the origin of lumbago in the kidneys or consider it a neuromyopathy of the dorsal lumbar region. In the first case, urotropin must act by its uric-acid-solvent and disinfectant properties; in the second case, we must assume either a direct action of the urotropin circulating in the blood upon the lumbar region or else an effect from the formaldehyde reabsorbed through the urinary mucosæ.

Weitlaner now makes it a routine practice to treat ordinary influenzas with a combination of urotropin (6-grain doses) with opium-ipecacuanha-sodium-salicylate in coffee, and considers this the best antiinfluenza treatment. He believes that urotropin is not without influence on the grippe itself.

